# Alternating Antibiotics Render Resistant Bacteria Beatable

**DOI:** 10.1371/journal.pbio.1002105

**Published:** 2015-04-08

**Authors:** Lauren Richardson

**Affiliations:** Public Library of Science, San Francisco, California, United States of America

By now we have all heard of the dangerous rise of antibiotic-resistant bacteria. While many search to identify new antibiotics and define their mechanisms, others wonder if there are better ways to use the antibiotics already available and approved for use in humans. This is doubly important given the rate at which bacteria adapt, as they will likely develop resistance to new antibiotics.

Techniques to better utilize current antibiotics include combination therapies and changes to dosing regimens. One example is sequential treatment, whereby bacterial infections are treated by alternating the use of different antibiotics. Sequential therapies have been used to treat cancer and have been successful in some instances in treating *Helicobacter pylori* infections. Using sequential therapies increases the optimization space of the treatment, meaning that there are exponentially more ways to design an effective treatment, and that increases the possibility of drugs cooperating in unanticipated ways.

In the study presented here, published in *PLOS Biology*, Ayari Fuentes-Hernandez, Jessica Plucain, Fabio Gori, Robert Beardmore, and colleagues demonstrate that two antibiotics known to act synergistically can be used in a specially designed sequential treatment to kill bacteria at dosages that, when the drugs are administered alone or in combination, cause rapid development of drug resistance and sustained bacterial growth.

The two drugs used were erythromycin (ERY) and doxycycline (DOX), both of which target the bacterial ribosome and act as inhibitors of translation. To help mimic more challenging clinical scenarios, the bacteria used in the in vitro model of infection contained a gene that encodes a multidrug efflux pump, the genomic amplification of which results in increased drug resistance to both drugs. Despite the presence and amplification of the efflux pump, the authors identified sequential treatments that killed the bacteria when monotherapies and combination therapies failed to do so.

The bacteria were cultured with medium and the antibiotic(s) for 12 h (one “season”), after which 1% of the culture was transferred to new medium with either fresh or different antibiotic(s). This process was continued for as many seasons as the experiment required; thus, for example, over eight seasons there would be 2^8^ (256) possible treatments, two of which are the monotherapies.

**Fig 1:**
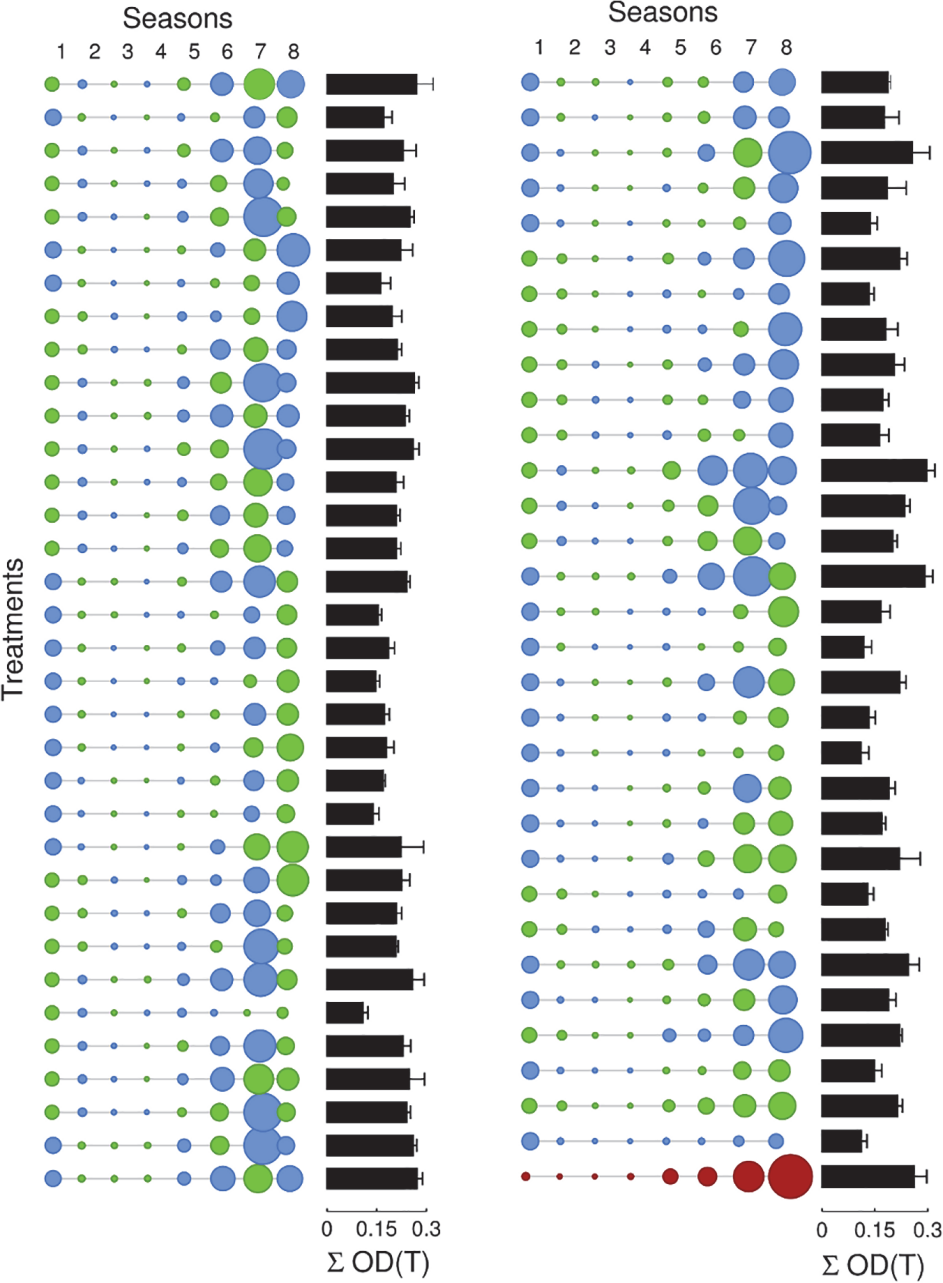
Can the sequential treatment of a bacterium with two antibiotics, in blue and green, have a greater efficacy than when the same antibiotics are combined into a cocktail, shown in red? The circles depict each of eight sequential “seasons,” with diameter reflecting population density in that season. Black bars at the right indicate the total cumulative bacterial population density, ΣOD(T).

First, the authors tested whether a sequential therapy could outperform monotherapy or combination therapy (Fig. 1). The combination therapy resulted in the greatest single-season inhibition (a nearly 95% reduction in cell density), but, by the end of the experiment, the cell densities rebounded, consistent with drug resistance. However, they did identify 16 sequential treatment patterns that cleared the bacteria. A follow-up experiment revealed that five of these treatments truly eliminated the entire bacterial population, whereas the other 11 had varying results. By contrast, neither the combination therapy nor either of the monotherapies achieved bacterial clearance.

The authors used whole-genome sequencing to determine genetic changes occurring during monotherapy versus sequential treatments and found that both treatments promoted the amplification of the multidrug efflux pump gene, as well as other known drug-resistance mutations. Therefore, the success of sequential treatments was not through averting or suppressing selection for drug resistance.

ERY and DOX act synergistically when given in combination, but, even at high doses, the combination treatment is not able to kill a bacterial population, as drug resistance still develops. Successful sequential therapy does not rely on drugs acting synergistically but on “collateral sensitivity.” Collateral sensitivity is when treatment with one drug sensitizes cells to treatment with a second drug. Conversely, cross-resistance is when treatment with one drug causes a decreased sensitivity to a second drug. One might expect that ERY and DOX would develop cross-resistance because they both select for the amplification of—and are exported out of the cell by—the same multidrug efflux pump. Undeterred by this assumption, the authors tested whether any sequential treatments of ERY and DOX exhibit collateral sensitivity. Cells were treated with one of the drugs for a number of seasons, after which they were treated for a single season with the second drug. When using ERY for multiple seasons followed by one season of DOX treatment, the authors saw a significant increase in cell density, consistent with cross-resistance. However, when they treated with DOX for multiple seasons followed by one season of ERY, they saw a significant reduction in cell density, consistent with a collateral sensitivity. Given these different results, this drug pairing is said to have a nonreciprocal collateral sensitivity.

While the exact mechanism behind the nonreciprocal collateral sensitivity of DOX and ERY is still unknown, the authors hypothesized that it is related to the different efflux efficiencies of the two drugs by the multidrug pump. When they deleted the gene for this pump, there was an approximately 95% decrease in the amount of ERY needed to halve the cell density, suggesting that ERY is very efficiently pumped out of the cells. For DOX, there was just a 77% decrease, indicating a slower efflux rate. Extended treatment with the highly efficiently pumped ERY likely exerts a stronger selection pressure on pump gene duplication—leading to a faster rise to prominence of the drug resistance allele in the population and making the cells cross-resistant to treatment with DOX. Conversely, extended treatment with the less efficiently pumped DOX potentially leads to a slower rate of resistance establishment, resulting in increased sensitivity to ERY.

While bacteria are masters at adapting to antibiotic challenge, this research suggests that there is a way to use this adaptation against them. The fluctuating environments created by well-designed sequential treatments can sensitize bacteria and render them susceptible to concentrations of antibiotics that would normally induce drug resistance and continued existence. Unfortunately, not all sequential treatments are equally efficacious. Extensive further work will be needed before sequential treatments make it in to the clinic, but this study demonstrates that they can be effective even when using drug doses below their maximal potency.
