# Correction: Alternating Antibiotics Render Resistant Bacteria Beatable

**DOI:** 10.1371/journal.pbio.1002254

**Published:** 2015-08-26

**Authors:** Lauren A. Richardson

The author’s name is spelled incorrectly. The correct name is: Lauren A. Richardson. The updated article citation is below.
